# Dietary Recommendations for Cyclists during Altitude Training

**DOI:** 10.3390/nu8060377

**Published:** 2016-06-18

**Authors:** Małgorzata Michalczyk, Miłosz Czuba, Grzegorz Zydek, Adam Zając, Józef Langfort

**Affiliations:** 1 Department of Nutrition & Supplementation, the Jerzy Kukuczka Academy of Physical Education in Katowice, Faculty of Physical Education, Mikołowska 72A, Katowice 40-065, Poland; m.michalczyk@awf.katowice.pl (M.M.); g.zydek@awf.katowice.pl (G.Z.); j.langfort@awf.katowice.pl (J.L.); 2 Department of Sports Training, the Jerzy Kukuczka Academy of Physical Education in Katowice, Faculty of Physical Education, Mikołowska 72A, Katowice 40-065, Poland; a.zajac@awf.katowice.pl

**Keywords:** altitude training, hypoxia, nutrition, cycling

## Abstract

The concept of altitude or hypoxic training is a common practice in cycling. However, several strategies for training regimens have been proposed, like “live high, train high” (LH-TH), “live high, train low” (LH-TL) or “intermittent hypoxic training” (IHT). Each of them combines the effect of acclimatization and different training protocols that require specific nutrition. An appropriate nutrition strategy and adequate hydration can help athletes achieve their fitness and performance goals in this unfriendly environment. In this review, the physiological stress of altitude exposure and training will be discussed, with specific nutrition recommendations for athletes training under such conditions. However, there is little research about the nutrition demands of athletes who train at moderate altitude. Our review considers energetic demands and body mass or body composition changes due to altitude training, including respiratory and urinary water loss under these conditions. Carbohydrate intake recommendations and hydration status are discussed in detail, while iron storage and metabolism is also considered. Last, but not least the risk of increased oxidative stress under hypoxic conditions and antioxidant supplementation suggestions are presented.

## 1. Altitude and Hypoxic Training

Cycling is an endurance sport discipline in which the athlete encounters significant training and competition loads and is often exposed to extreme environmental conditions. Therefore, in cycling numerous performance-enhancing nutritional and physiological aids are used to improve the efficiency of the cardio-respiratory system. One of the legal and natural performance enhancing methods used in cycling includes altitude training, which significantly improves the cardio-respiratory potential.

The concept of altitude or hypoxic training is a common practice in cycling not only for improving sport performance at sea level but also at moderate altitude [1,2,3]. Cyclists often compete in races (e.g., Tour de France, Giro d ’Italia and Vuelta a España) at moderate altitudes (from 1000 to 3000 m a.s.l); what requires a specific adaptation to a hypoxia environment. At these conditions increasing altitude and the consequent reduction of air density is beneficial from the aerodynamic perspective [4], but on the other hand acute hypoxia deteriorates exercise performance [5,6]. In particular, the maximal aerobic workload that can be sustained during exercise involving large muscle groups (e.g., cycling) is considerably lower in hypoxia compared with normoxia. The origin of human performance limitation in hypoxia is attributed to a decrease in VO_2max_. Dempsey and Wagner [7] observed that each 1% decrement in SaO_2_% below the 95% level approximates to a 1%–2% decrement in maximal oxygen uptake (VO_2max_). Diminished VO_2max_ in hypoxia is accompanied by a lowered O_2_ partial pressure in arterial blood (PaO_2_), which reduces O_2_ delivery to tissues and negatively affects muscle metabolism and contraction [8,9], leading to so-called peripheral fatigue.

After 40 years of altitude training, several strategies of such training regimens have been proposed, like “live high, train high” (LH-TH), “live high, train low” (LH-TL) or “intermittent hypoxic training” (IHT). Each of them combines the effect of acclimatization and different training protocols, which requires specific nutrition [3,10,11]. These nutrition concepts are due to different time of exposure to hypoxia at rest and different combinations of training under hypoxia and exposure to these conditions. In the LH-TH and LH-TL methods the acclimatization depends primarily on the iron status of the body, as well as on the maintenance of acid-base and energy equilibrium, what can significantly influence erythropoiesis. In the IHT method the dietary recommendations for athletes are less strict, and concentrate on pre-, mid- and post training unit nutrition. The specific demands of IHT relate to greater delivery of carbohydrates and better hydration.

According to the first mentioned method, athletes live and train in a natural hypobaric hypoxic environment at moderate altitude for a few weeks. Chronic exposure to moderate altitudes (2000–3000 m) improves oxygen transport capacity by enhancing erythropoietin secretion and the consequential increase in total hemoglobin mass [12,13]. This adaptive change improves maximal oxygen uptake (VO_2max_) and enhances physical performance [14]. Chronic exposure to hypoxia may also reduce the energy cost of exercise at sea level by more efficient cellular metabolism [13]. The mechanism responsible for the decreased energy cost of exercise at sea level after altitude training is related to the increase of ATP production per molecule of O_2_ utilized [15], and/or a decreased ATP breakdown during muscular contractions [16]. These adaptive changes can be seen already after 3 to 4 weeks of exposure to moderate altitudes, but the main factor limiting the effectiveness of the LH-TH concept is that many athletes cannot maintain the required training intensity while staying at an altitude for a longer period of time, and consequently decrease their level of endurance and technical abilities [11]. In response to this weak point of LH-TH method, the LH-TL method was proposed by Levine and Stray-Gundersen [10]. The LH-TL protocol allows athletes to “live high (2000–3000 m)” for altitude acclimatization while “training low” (below 1000 m) for the purpose of replicating low-altitude training intensity and oxygen flux, thereby inducing beneficial metabolic and neuromuscular adaptations [11]. In this method athletes can live in a natural hypobaric environment, or use special technology based on nitrogen dilution or oxygen filtration, to simulate physiological adaptive changes by creating a normobaric hypoxia environment [17,18]. However, the current results of research on the efficacy of the LH-TL method are controversial. There are some studies which support the performance enhancing effects of LH-TL training on endurance performance and aerobic capacity [1,17,18], and those that do not confirm such effects [19,20].

Recently, significant attention in sport sciences, as well as in competitive cycling has been given to IHT, which theoretically, may cause more pronounced adaptive changes in muscle tissues in comparison to traditional training under normoxic conditions [21]. In this method, athletes live under normoxic conditions and train in a natural hypobaric or simulated normobaric hypoxic environment. The improvement in sea-level performance and an increase in VO_2max_ after IHT cannot be explained by changes in blood variables alone, but is also associated with non-hematological adaptive mechanisms [3]. The results of our previous studies [3,13] and other well-controlled studies [22,23] indicate that the improvements in aerobic capacity and endurance performance are caused by muscular and systemic adaptations, which are either absent or less developed after training under normoxia. These changes include increased skeletal muscle mitochondrial density, elevated capillary-to-fiber ratio, and increased fiber cross-sectional area [24,25].

Acute and chronic exposure to hypoxia induces serval metabolic consequences in the body and combined with physical exercise under hypoxic conditions presents an enormous challenge for athletes [26,27,28,29]. A significantly lower oxygen concentration in the blood, forces the body to produce the energy primarily from other substrates than in normoxia [30]. The athlete’s body needs 2–3 weeks to adapt to the low level of oxygen, or else they feel fatigue, headaches and a decrease in appetite [31]. An appropriate nutrition strategy can help athletes achieve their fitness and performance goals in this unfriendly environment.

In this review the physiological stress of altitude exposure and training will be discussed, with specific nutrition recommendations for athletes training under such conditions [32]. However, there is little research about the nutrition demands of athletes who train at moderate altitude (2000–3000 m) [33,34]. Only in a few studies the authors assessed the nutritional habits of athletes training under hypoxia [31,32]. The data and nutrition recommendations in this review relate primarily to cycling, but they can be applied to other aerobic endurance sport disciplines such as the triathlon, Nordic skiing or the biathlon.

## 2. Body Composition during Altitude Training

There are some evidences that body composition of athletes exposed to altitude may be significantly changed after training. First of all, during acute exposure there may be a slight reduction in total body mass due to increased respiratory and urinary water loss. However, chronic oxygen deprivation observed initiates many physiological changes, with the most prominent changes being loss of body mass and protein stores especially at high altitudes (above 5000 m) [35], as well as fat content [36,37]. However, chronic exposure to moderate altitudes has also been reported to be an important factor in skeletal muscle atrophy [3,38]. Changes in fat and muscle mass in athletes may be a consequence of increased basal metabolic rate [39], as well as increased training loads [40] in combination with decreased caloric intake. However, Kayser [40] stated that people can prevent body composition changes by maintaining an adequate caloric intake, when they stay below 5000 m. This fact is very meaningful to athletes because altitude training camps are typically located at elevations from 2000 to 3000 m. Therefore proper nutrition strategy is a key factor determining the effectiveness of altitude training (LH-TH). For example Svedenhag *et al.* [41] and Gore *et al.* [42] reported insignificant differences in body composition in endurance athletes (runners and cyclists) after few weeks of altitude training conducted at moderate altitude (2000 and 2700 m). According to the authors, these athletes experienced this effect despite proper nutrition and adequate hydration.

On the other hand, Etheridge *et al.* [43] indicated that breathing normobaric hypoxic air (FiO_2_ = 12%) in a post-absorptive state did not modify muscle protein synthesis at rest, but rather blunted the increase in protein synthesis induced by exercise. Acute hypoxia (intermittent hypoxic training) was also shown to inhibit muscle protein synthesis [44] primarily by inhibiting mechanistic target of rapamycin complex 1 (mTORC1) via activation of the AMP-activated protein kinase (AMPK) [45].

## 3. Hydration during Altitude Training

The maintenance of proper fluid balance during cycling training and competition is a key factor determining sport performance. However, creating a successful nutrition strategy, especially in a hot and humid environment is a great challenge. Proper hydration seems even more important for athletes training at altitude. Within the first few days at altitude, there is a tendency toward dehydration due to increased respiratory water loss by enhanced ventilation [26], and increased urinary water loss secondary to downregulation of the renin-angiotensin-aldosterone hormone mechanism [46]. Therefore, at moderate altitudes up to 4000 m respiratory water loss may be increased to 1900 mL per day in men [39] and 850 mL per day in women [47]. Besides, urinary water loss may increase up to 500 mL per day [48]. Cyclists during altitude training need to maintain fluid balance through regular hydration, in conjunction with daily workouts as well as during the restitution period of the day. Fluid intake in the form of water, isotonic carbo-electrolyte drinks, and juices should be increased even up to 7 L per day to insure adequate hydration [38,49]. Saris [38] reported that during the Tour de France mountain stages of the race, several cyclists drank more than 10 L of fluid per day. On the other hand cyclists must be cautious not to overhydrate their bodies, as this may hinder the adaptive processes and decrease performance. According to the authors of this review regular monitoring of body mass and urine osmolality during altitude training is absolutely necessary. This relates to the range altitude used for training purposes (2000–3500 m) and training loads. Athletes and coaches must take into consideration the fact that diuretic drinks like coffee and tea, as well as energy drinks with caffeine, can increase the diuretic effect but on the other hand can help increase the intensity of exercise and reduce the perception of fatigue.

The natural high altitude environment in addition to low oxygen concentration is often accompanied by low air temperature. To cope with these unfavourable conditions, and to maintain optimal body temperature, athletes must increase their basic metabolic rate to prevent hypothermia [31,39,40]. The acclimatization to hypoxia may induce different molecular adaptive responses. Decreased oxygen concentration under hypoxic conditions causes the muscle cells to accumulate large amounts of multi gene transcription protein like HIF-1 (Hypoxia Inducible Factor), which is known to regulate the synthesis of EPO (Erythropoietin) and VEGF (Vascular Endothelial Growth Factor), proteins required for erythropoiesis and angiogenesis. HIF-1 also regulates transcription oxidative pathway enzymes like pyruvate dehydrogenase (PHD), increases activity of lactate dehydrogenase (LDH), inhibits mitochondrial biogenesis, and activates the transcription of genes encoding glucose (GLUT1) and lactate MCT4 transporters as well as glycolytic enzymes. Because of lower oxygen tension, energy synthesis, both at rest and during exercise is mainly supplied by the glycolysis pathway. In short acute hypoxia exposure, lactate (La) concentration for submaximal exercise is higher than in normoxia, without peak La value changes. Gore *et al.* [50] showed an almost 10% improvement of efficiency during submaximal exercise after altitude acclimatization. Hoppler [51] indicated that training in hypoxia results in an increase of phosphofructokinase (PFK) mRNA, an enzyme which is involved in the glycolytic pathway, HIF-1mRNA, myoglobin mRNA and VEGF mRNA as well as mitochondria density [51,52] which may lead to increased oxidative metabolism. However, Lundby *et al.* [53] did not confirm that 8 weeks of exposure to hypoxia increases muscular VEGF m RNA expression and capillary density.

Energy expenditure in athletes who train and live at high altitude could be 2.5–3 times higher than at sea level [31,39,54]. However, during the Tour de France, elite cyclists recorded a 3.6–5.3 higher energy expenditure than the resting metabolic rate [34]. Duc found that energy cost during ski mountaineering racing at high altitude increases by approximately 15% [55]. Lack of critical macronutrients like carbohydrates, fats and proteins can enhance hypothermia, decrease metabolic rate, disturb optimal performance and decrease body mass [30,56]. It is believed that food intake may limit exercise performance in cycling events at altitude like the Tour de France, and the main factors limiting this performance include the ability to maintain energy balance and muscle mass [34].

Carbohydrates and protein must be delivered during high altitude physical activity to maintain body weight, replenish glycogen stores, and provide adequate protein to build and repair tissue [32,39]. Fat intake should also be sufficient to provide essential fatty acids and fat-soluble vitamins, and to contribute energy for weight maintenance. During an expedition to Mt. Everest Reynolds *et al.* [31] observed a significant decrease in energy consumption in climbers at increasing altitude but no changes in total carbohydrate, fat and protein consumption. Between climbers it is commonly assumed that there is a natural tendency to increase the consumption of carbohydrate intake at higher altitudes [57]. Carbohydrate consumption before exercise in hypoxia alleviates some of the negative symptoms of high altitude, like decreased appetite, less oxygen saturation and less ventilation [58]. Golja *et al.* [58] showed that carbohydrate consumption 40 min prior to acute hypoxia exposure increases ventilation and oxygen saturation, thereby oxygen delivery to the tissues.

Vitamin and mineral supplements are not needed for athletes at high altitude if adequate energy to maintain body weight is consumed from a variety of foods [59,60,61]. On the other hand, athletes who restrict energy intake due to lack of appetite, eliminating one or more food groups from their diet because of intolerance, or consuming unbalanced diets with low micronutrient density may require additional supplements.

## 4. Dietary Carbohydrate Intake Recommendations

Athletes training or competing at high altitude dramatically increase the rate of energy expenditure compared to normoxia [54]. It is critical to obtain sufficient energy intake to support total energy requirements including those for muscle activity but also for tissue maintenance and repair. Athletes training and competing under such conditions should make a conscious effort to eat at frequent intervals. It is important that athletes and their coaches understand how appropriate energy intake and energy substrate utilization enhance mental and muscle function. It is well known that the higher the exercise intensity, the greater the amount of carbohydrates used as fuel for working muscles. For athletes like road cyclists who train with extremely high loads for several hours a day, the most important source of energy for working muscles includes carbohydrates [62,63,64,65]. These substrates need less oxygen than fats and protein to be metabolized for ATP resynthesis. Consumption of adequate amounts of carbohydrates is especially important where cold stress and shivering occurs [66].

Athletes should provide the right amount of carbohydrates before, during and after exercise at high altitude [49]. It is absolutely clear that low pre-exercise muscle glycogen stores result in reduced exercise intensity [67]. A cyclist’s diet during altitude training and competition should contain more than 60% CHO with one-third coming from liquid CHO due to reduced hunger at altitude [33]. Brouns [49] reported that during exhausting training sessions at altitude CHO intake increased up to 80% of consumed calories per day. It is suggested that cyclists consume 12–13 g of CHO per kg of body mass per day [33], what was confirmed by Rehrer’s research [34], which presented values of 12.9 g CHO/kg of body mass per day in the Tour de France race. It is suggested that in endurance athletes like cyclists, inadequate carbohydrate consumption before and during training or competitions at high altitude may result in a reduction of exercise capacity. Adequate carbohydrate consumption before exercise increases glycogen stores in the muscle and liver. Eventually, insufficient intake of carbohydrates at high altitude may also cause low blood glucose levels, which leads to central fatigue [68]. Sufficient carbohydrate consumption after training or competition provides quick glycogen resynthesis, reduced muscle soreness and enhanced muscle recovery [63].

Athletes who train in hypoxia must consume carbohydrates to provide quickly and easily assimilated sources of energy for muscle and brain, between meals and during exercise, to optimize glycogen stores before and after exercise, and to enhance muscle recovery after physical activity. Additionally the carbohydrates must provide the energy to maintain blood glucose level between the main meal and during exercise. According to the International Society of Sports Nutrition, carbohydrates should provide 55%–65% of total caloric intake [69]. These authors indicate that while determining the optimal amount of carbohydrate intake the conversion to body weight should be applied. The carbohydrate intake recommendation for endurance trained athletes range from 7 to 10 g/kg of body mass per day [62]. Road cyclists after very intensive high altitude competition, which lasts from 4 to 6 h, should consume up to 12 g of carbohydrate per kilogram of body mass per day [62]. Some authors suggest that the average amount of carbohydrate which enhances cyclists performance is 300–400 g for meals consumed 3–4 h before exercise [70,71].

It is very difficult to deliver that quantity of carbohydrates in the form of traditional meals. Taking this into consideration, athletes consume a large part of their carbohydrates in the form of supplements, usually liquid form. In addition during high altitude training or competitions athletes suffer from appetite suppression and other gastrointestinal problems, which may contribute to inadequate energy intake [64]. They often suffer from weight loss, especially muscle mass, which negatively affects endurance and strength capacity [72]. The amount of consumed carbohydrates is not the only important factor determining the delivered energy during exercise. Attention should be paid to other factors like meal temperature, osmolality and exercise intensity, as these factors determine gastric empting and intestines absorption [64,73]. However, in order to calculate the individual carbohydrate recommendations for high altitude, other factors like gender, body weight and training status should also be considered [64].

Between regular meals or training sessions conducted at altitude, athletes should consume high-carbohydrate, nutrient-rich snacks, which are a good alternative energy supply [54]. All these recommendations should be adjusted to individual requirements of athletes. It is also important to choose recovery meals that contain various components besides carbohydrates. Several authors suggest that carbohydrates consumed with proteins after exercise aid glycogen resynthesis [74].

Road and off-road cyclists often modify their diets 1–2 days before competition, using the carbohydrate loading procedure to enhance muscle glycogen [63]. This procedure assumes that to achieve muscle glycogen super compensation, ingestion of 10 g of carbohydrate per kilogram of body mass per day is recommended [54]. It is critical for athletes to consume different kinds of carbohydrates. During and immediately after exercise, carbohydrate products with a high glycaemic index should be preferred. They can include glucose or disaccharides derived from liquid, semiliquid and solid foods, like sport drinks and fruit bars [64]. On the other hand, during main meals, athletes should consume rather complex low glycaemic carbohydrates, derived from solid foods like cereals and grains, breads, vegetables, fruits and legumes [64]. To optimize muscle glycogen resynthesis after training or competition, it is recommended that cyclists consume 1.37–1.72 g CHO∙kg^−1^·h^−1^ [33] or 1.5 g/kg^−1^·h^−1^ carbohydrates, for the first 4 h after exercise [62]. Post training and post competition meals should contain carbohydrate rich foods and fluids with a high and medium glycaemic index [62]. When less than 1 g of carbohydrates per kilogram per hour is ingested, the meal should also contain proteins to provide a higher rate of glycogen synthesis [74].

In addition to suitable calories and carbohydrate consumption, athletes who train under conditions of inadequate oxygen concentration should provide adequate amounts of B vitamins like folic acid, vitamin B_12_ and iron [60,75]. A healthy individually balanced diet should supply most of the needed macronutrients, which are necessary to produce haemoglobin, but otherwise some vitamin and mineral supplementation should be considered [69].

## 5. Antioxidants

Performing endurance training at high altitude requires an increased demand not only for energy but also for vitamins and minerals [76]. In the past decade or so, we have observed increased antioxidant supplementation in competitive athletes, especially in endurance sport disciplines, such as road cycling, long distance running and Nordic skiing [77,78,79]. However numerous controversies have risen about the benefits of antioxidant supplementation in athletes. Some authors argue that antioxidants can protect muscle cells against oxidative damage [77,80], while others argue the contrary [78,81,82,83]. Many authors suggest that vitamin and mineral supplementation should be considered by athletes before exposure to high altitude, because under those conditions muscle cells release a lot of free radicals which are highly reactive [28]. During typical cycling training at high altitudes, were hypoxia occurs, muscle cells release large amounts of reactive oxygen/nitrogen species (RONS), which can damage cell lipids, proteins and DNA structure causing cell dysfunction and, eventually, apoptosis [84,85,86]. Oxidative damage of polyunsaturated lipid membranes seems especially harmful, as it results in a decrease of membrane fluidity, compromised integrity, and inactivation of membrane bound protein receptors and enzymes [79,84].

If muscle cells produce large amounts of RONS, they can provide significant oxidative stress [85,87]. In essence oxidative stress presents an imbalance between production and degradation of free RONS [88,89]. Such conditions may lead to a physiological imbalance in cells and tissues and cause inflammation, overloading or even overtraining. However it is still unclear whether oxidative stress is harmful to athletes [82,86]. RONS play an important role in the regulation of the body’s immune system, counteract tissue insulin resistance and cell signalling [90]. Researches generally confirm that in athletes oxidative stress can promote mitochondria biogenesis, cellular growth, proliferations and increased antioxidant enzymes gene expression [82,83]. During typical endurance training or competitions the primary source of RONS includes the mitochondrial respiratory chain, where almost 2% of all oxygen consumption is converted to damaging superoxide radicals [91]. A different source of RONS includes xanthine oxidase reactions during ischemia/reperfusion—transient tissue hypoxia conditions, catecholamine auto oxidation and lactic acid reactions [79,92]. Scientists also discovered that especially haem proteins like haemoglobin or myoglobin in the Fenton reaction can generate highly reactive hydroxyl radicals, while during auto oxidation of those proteins superoxide radicals can be produced [90]. These states are very frequent during road cycling training in normoxia conditions, as a response to muscle damage [80,93]. These processes are even intensified in athletes training under hypoxia [85,87]. To protect against oxidative damage muscle cells contain complex endogenous cellular defence mechanisms [92]. There are several enzymes and small scavengers which are involved in converting or removal of RONS. Antioxidant enzymes like superoxide dismutase (SOD), glutathione peroxidase (GPX), catalase (CAT) or glutathione reductase (GR) form the first line of defence against free radicals [79,92]. The second line of defence includes small scavengers like vitamin E and vitamin A, located in the cell membrane [84] as well as glutathione and vitamin C located inside the cells [92].

Recently numerous research projects regarding antioxidant supplementation in athletes have been conducted [94,95]. Most authors used typical supplements like vitamin C, A and E [94,96] or their combinations [80,95,97]. Some authors confirm positive effects of antioxidants/vitamin C supplementation [98,99]. Maxwell and Aschton suggest that pre exercise vitamin C supplementation attenuates the level of exercise induced free radicals and reduces exercise induced muscle damage [98]. Bryant confirms that supplementation with 400 IU of vitamin E per kg of body mass is more effective than 1 g per day vitamin C supplementation or a combination of 1 g vitamin C per day plus 200 IU vitamin E per kg, to reduce lipid peroxidation level in trained cyclists at sea level [99]. However Purkayastha [100] suggests that in men 400 mg/day vitamin E supplementation prevented stress at moderate altitude (3700 m). On the other hand supplementation of 400 IU vitamin E per day, did not significantly affect markers of oxidative stress associated with increased energy expenditure at high altitude [101]. In recent years, growing evidence indicates that exercise-induced production of reactive oxygen species serves as a signal to promote the expression of numerous skeletal muscle proteins, including antioxidant enzymes, mitochondrial proteins, and heat shock proteins [102,103]. Furthermore, two recent reports indicate that antioxidant supplementation with high levels of vitamins E and C (*i.e.*, 16 times higher than the recommended dietary allowance for adults) can blunt the training adaptation to exercise under normoxia [79,83].

In another study, Bentley showed that acute supplementation of trained cyclists 4 h prior to an exercise trial with antioxidant pine bark extract increased maximal oxygen uptake and extended time to exhaustion [104]. Nieman used quercetin supplementation as a form of an antioxidant, and showed improved exercise performance and increased muscle mitochondrial biogenesis [105]. Most well-controlled studies report no attenuating or even negative effect of antioxidant supplementation on oxidative stress markers [79,80,81,82,83]. Some authors suggest that antioxidant supplementation may promote muscle damage and cause longer recovery [81,106].

Numerous recent research projects have concentrated on the beneficial biological effects of antioxidants contained in vegetables and fruits that can currently be identified and measured [107,108,109]. In contrast to antioxidant supplements, plant foods contain many different kinds of antioxidants, like vitamin E and C, carotenoids and other phytochemicals that can act as synergists [110,111].

Most researches have confirmed that cyclists who undertake very high training loads, either living and/or training at moderate to high altitudes, or who participate in ultra-endurance competitions have an antioxidant imbalance [85,87,112]. There are reports indicating that cyclists who train under intermittent hypoxia conditions show lower plasma antioxidant levels [85]. It seems that under such circumstances the athletes can benefit from natural antioxidant supplementation. Considering the present state of knowledge, it seems that the best natural source of antioxidants comes from a diet full of fresh vegetables, fruits and flavours [107,113]. A well balanced diet full of natural antioxidants can minimize the level of oxidative stress produced during high volume and high intensity training [114]. While training at altitude, athletes, should consume high amounts of different kinds of micellar lyophilized fruit like blueberry, acai berry, goy berry, red grapes, raspberry, orange, papaya, blackcurrant, cherry, kiwi, strawberry, red grapes, mango, melon, grapefruit and lemon [115,116]. Those fruits are rich in naturally occurring vitamin C, carotenes, polyphenols and many others phytochemicals [108,110,113]. A cyclist’s diet should also contain large amounts of vegetables, especially lyophyilizate products, like tomato, carrot, spinach, beetroot, broccoli, parsley, avocado, which are naturally full of antioxidants, such as vitamin A, vitamin C, carotenes, glutathione, resveratrol and quercetin [105,108,109]. In additions to vegetables and fruit, it is suggested that endurance athletes also consume flavouring, especially cloves, cinnamon, oregano, curcumin seed, cumin seed, basil, curry powder, pepper, with many bioactive compounds like flavonoids and anthocyanins which may directly or through their metabolism affect the total antioxidant capacity of plasma and tissue [87,113,117].

## 6. Iron Storage

Apart from antioxidant vitamins and phytochemicals, minerals like copper, zinc, manganese, selenium and iron, which act as cofactors of antioxidant enzymes, are very important in an athlete’s diet [92,114]. Iron status in particular should be at a high level before attempting altitude training. In addition to the previously mentioned role of iron in the production of red blood cells, it plays an important role in the antioxidant defence not only as an antioxidant microelement but also because appropriate supply of oxygen to the working muscles depends indirectly on the level of iron [60].

As a result of acclimatization to altitude due to an increase in erythropoiesis, a decline in iron storage in the blood is observed [118]. In studies conducted by Roberts and Smith [119] and Pauls *et al.* [118], a significant reduction in the concentration of ferritin in the blood at altitudes above 2000 m was observed. Low levels of ferritin and iron in the blood can impair the increase in haemoglobin concentration in athletes exposed to hypoxia. It should also be noted, that in early research with altitude training [120,121,122,123] scientists did not control the concentration of iron and ferritin in the blood. The lack of this data makes it difficult to explain the improvement of aerobic capacity of the blood after altitude training. This interpretation is supported by data obtained by Stray-Gundersen *et al.*, [27], which show an improvement in erythropoiesis at altitude in case of low concentration of ferritin in the blood. These authors also reported, a lack of changes in haematological variables during altitude training when serum ferritin was less than 30 ng·mL^−1^ in men and 20 ng·mL^−1^ in women. The results of these studies have provided evidence that the level of ferritin must be monitored regularly, before and during altitude training. Additionally, in many studies athletes were supplemented with iron (even up to 100 mg daily) during altitude training to prevent potential anaemia from occurring.

Due to the slow replenishment, iron deficit should be completed several months before high altitude training [28]. The best source of iron is red meat like beef, offal and seafood. Vegetarian cyclists to provide appropriate amounts of iron should consume higher amounts of soya beans, beans, and green vegetables like parsley, broccoli and sprout. Unfortunately, the iron of those products due to significant contents of fibre is poorly absorbable. Grains, seeds and nuts are a very good source of other minerals mentioned above.

## 7. Vitamin D

The identification of the vitamin D receptor in the heart and blood vessels raised a possibility of potential cardiovascular effects of vitamin D [124,125], and thereby most likely on aerobic exercise, which is known to induce cardiovascular changes associated with marked increases of aerobic power and endurance performance [126]. There is evidence that vitamin D causes vascular relaxation by suppressing the renin-angiotensin-aldosterone system [127,128] and improves cardiomyocyte contractility [129] that may create physiological conditions for more efficient skeletal muscle oxygenation. Moreover, some data indicate that vitamin D is necessary for maintenance of skeletal muscle structural integrity and function [130]. This study raises a question if vitamin D supplementation may take part in protection of skeletal muscle against atrophic changes seen under hypobaric hypoxia conditions [39,131]. Findings confirming the high prevalence of vitamin D deficiency in the general population, as well as in athletes [132], and a significant decrease of serum vitamin D level in alpinists after their return from mountaineering expeditions (14 days, 3200–3616 m above sea level) [133] suggests that vitamin D supplementation should be considered in athletes who stay at high altitude. Such an assumption is further supported by the fact that the conversion of 25(OH)D into1,25(OH)2D_3_ within the kidney by the enzyme 1alfa-hydroxylase is O_2_-dependent and hypoxic conditions induce enzyme inhibition [134].

At altitude the intensity of UV radiation increases, creating favorable conditions for vitamin D synthesis, but to take full advantage of these environmental conditions the athlete’s body has to be exposed to sunlight. It seems logical that during the summer months, when light clothing is worn during training, vitamin D synthesis should be increased, as opposed to colder parts of the year, when the body is usually fully covered. Unfortunately there are no data regarding this topic.

## 8. Alkalizing Agents

Considering the course of adaptive changes to altitude training supplementation with alkalizing agents (beta-alanine and bicarbonate) seems unjustified. One of the main adaptive changes induced by hypoxia training includes the increased buffering of the blood and muscle tissues. One of the acute as well as chronic adaptive changes to hypoxia is hyperventilation, with the objective of maintaining vacuole PO_2_ at a steady level. Do to altitude hyperventilation, an increased diffusion of vacuole CO_2_ occurs what causes the so called respiratory alkalosis. This response may improve the buffering capacity of tissues through a decrease in pH and an increased excretion of bicarbonates through the kidneys. An increased buffering capacity can significantly improve high intensity exercise potential. This has been confirmed by research of Mizuno *et al.*, [135], in which the authors observed a 6% increase of buffering capacity in the gastrocnemius muscle, as well as a 17% increase in the time to exhaustion performed on a treadmill in elite Nordic skiers living at an altitude of 2100 m and training at 2700 m for 2 weeks. According to these training concepts, cyclists exposed to hypoxia improved their muscle buffering capacity by 18%, and afterwards improved their results significantly during a specific cycling test performed under normoxic conditions [1]. The mechanism responsible for increased buffering capacity of muscle tissues under conditions of hypoxia is not fully examined, yet most likely it may occur do to the buffering properties of phosphocreatine and the concentration of muscle proteins [135]. On the other hand the improvement of blood buffering capacity is related to the higher concentration of hemoglobin and bicarbonates [136].

## 9. Conclusions and Recommendations

Considering the above reviewed data, it is not easy to create specific nutrition recommendations for cyclists and other endurance athletes training at altitude. This stems from a lack of well-controlled research under these conditions in competitive athletes. Precise dietary recommendations are difficult because of the great range of altitude at which exercise and exposure take place, which varies from 2000 to 3500 m. Additionally, athletes use different volume and intensity of exercise, depending on their sports level and part of the season. The most important aspects of nutrition strategy for altitude training for competitive athletes include proper hydration and optimal energy balance. According to the authors, it is difficult to recommend a strictly defined intake of fluids, as it is dependent on several variables such as range of altitude, air temperature, humidity, and most of all, the entire training load. Thus fluid intake should be monitored on a daily basis through body mass measurement and urine osmolality. Several reports are available that indicate an intake of close to 10 L daily for cyclists undertaking heavy training loads at moderate altitude. Monitoring of fluid intake is also of great significance because of the threat of over hydration which has shown effects of decreased adaptation to hypoxia. Another important aspect of nutrition during altitude training includes increased energy intake through the consumption of greater amounts of CHO. One must also consider the fact that exposure to altitude suppresses hunger and appetite, what can lead to a negative energy balance. A high carbohydrate diet is recommended for athletes exercising intensively at altitude with the upper daily range of CHO consumption close to 12 g/kg. A well-balanced diet with an increased caloric intake should provide a sufficient amount of all antioxidants, so we do not recommend additional supplements which could hinder the adaptive processes related to aerobic endurance. Antioxidant supplementation should be considered only when natural food sources such as fruits and vegetables are not available at altitude. One of the more significant elements of altitude nutrition relates to the monitoring of iron, for which intake needs to amount to at least 100 mg/day. A deficit of iron may disturb erythropoiesis. Despite the increased UVB radiation from sunlight, it is recommended to supplement athletes training at altitude with up to 4000 IU/day of vitamin D, especially in the winter months of the year.

The presented data clearly shows great deficits in research related to nutrition and dietary recommendations for competitive athletes training at altitude. General guidelines for altitude nutrition can be proposed, but specific recommendations require further, well controlled research.
